# Promoter hypermethylation analysis of host genes in cervical intraepithelial neoplasia and cervical cancers on histological cervical specimens

**DOI:** 10.1186/s12885-023-10628-5

**Published:** 2023-02-20

**Authors:** Liye Shi, Xue Yang, Ling He, Chunying Zheng, Zhen Ren, Juweria Abdisamad Warsame, Suye Suye, Lei Yan, Haiyi Cai, Xiao Xiao, Chun Fu

**Affiliations:** 1 Department of Obstetrics and Gynecology, The Second Xiangya Hospital of Central South University, Changsha, China; 2 Department of Clinical Medicine, Harbin Medical University, Harbin, 150081 China

**Keywords:** Cervical cancer, Cervical intraepithelial neoplasia, DNA methylation, GynTect® assay, High-risk human papillomavirus

## Abstract

**Background:**

DNA methylation is an essential factor in the progression of cervical intraepithelial neoplasia (CIN) to cervical cancer. The aim was to investigate the diagnostic value provided by methylation biomarkers of six tumor suppressor genes (*ASTN1, DLX1, ITGA4, RXFP3, SOX17* and *ZNF671*) for cervical precancerous lesions and cervical cancer.

**Methods:**

The histological cervical specimens of 396 cases including 93 CIN1, 99 CIN2, 93 CIN3 and 111 cervical cancers were tested for methylation-specific PCR assay (GynTect®) of score and positive rate. Among them, 66 CIN1, 93 CIN2, 87 CIN3 and 72 cervical cancers were further used for paired analysis. A chi-square test was used to analyze the difference of methylation score and positive rate in cervical specimens. The paired t-test and paired chi-square test were for analyzing the methylation score and positive rate in paired CIN and cervical cancer cases. The specificity, sensitivity, odds ratio (OR) and 95% confidence interval (95% CI) of the GynTect® assay for CIN2 or worse (CIN2 +) and CIN3 or worse (CIN3 +) were evaluated.

**Results:**

According to the chi-square test trend, hypermethylation increased with severity of the lesions as defined by histological grading (*P* = 0.000). The methylation score above 1.1 was more common in CIN2 + than in CIN1. The DNA methylation scores in the paired groups of CIN1, CIN3 and cervical cancer were significant differences (*P* = 0.033, 0.000 and 0.000, respectively), except for CIN2 (*P* = 0.171). While the positive rate of GynTect® in each paired group had no difference (all *P* > 0.05). The positive rate of every methylation marker in the GynTect® assay showed differences in four cervical lesion groups (all *P* < 0.05). The specificity of GynTect® assay for detection of CIN2 + /CIN3 + were higher than high-risk human papillomavirus test. With CIN1 as a reference, the positive status of GynTect®/*ZNF671* were significantly higher in CIN2 + : odds ratio (OR) 5.271/OR 13.909, and in CIN3 + : OR 11.022/OR 39.150, (all* P* < 0.001).

**Conclusion:**

The promoter methylation of six tumor suppressor genes is related to the severity of cervical lesions. The GynTect® assay based on cervical specimens provides diagnostic values for detecting CIN2 + and CIN3 + .

**Supplementary Information:**

The online version contains supplementary material available at 10.1186/s12885-023-10628-5.

## Novelty & impact statements

Detection of DNA methylation may be helpful to identify cervical cancer and advanced cervical intraepithelial neoplasia (CIN). The majority of DNA methylation studies focused on scraping specimens for cervical cancer screening. This study of promoter methylation (*ASTN1*, *DLX1*, *ITGA4*, *RXFP3*, *SOX17* and *ZNF671*) by the GynTect® assay was detected in histological cervical specimens. According to the chi-square test trend, hypermethylation increased with the severity of the lesions as defined by histological grading. The GynTect® test based on cervical specimens has good diagnostic values for detecting CIN2 + and CIN3 + .

## Background

Cervical cancer is the most commonly diagnosed cancer in 23 countries and is the leading cause of cancer death in 36 countries [1]. Preventing progression of cervical lesions is a pivotal step in reducing incidence of cervical cancer. In the process of cervical intraepithelial neoplasia (CIN) developing into cervical cancer, CIN can be divided into "productive" and "transforming" according to its risk of cervical cancer [2, 3]. In addition to high-risk human papillomavirus (hrHPV) persistent infection, DNA methylation of host-cell genes is a common event in cervical carcinogenesis [3]. DNA methylation is an essential factor in promoting the progression of CIN to cervical cancer [4, 5]. Although CIN3 is morphologically regarded as a severe precancerous lesion, it represents a heterogeneous disease [4]. Therefore, DNA methylation detection may aid in the early detection, risk stratification and treatment response of cancer.

DNA methylation occurs in both HPV and host cellular genomes during all stages of cervical carcinogenesis [6–10]. The hypermethylation of the promoter of tumor suppressor genes is an important reason for the rapid proliferation of cervical cancer cells. The methylation level of more than 100 host genes is related to cervical cancer. Studies have found that *CADM1, CDH1, DAPK1, EPB4L3, FAM19A4, MAL, PAX1, PRDM14* and *TERT* are among the most commonly methylated genes in cervical cancer and precancerous lesions [2, 11–15]. At present, host cell DNA methylation analysis is mainly used for preliminary triage of hrHPV-positive women to detect cervical cancer and advanced CIN [8]. As histology is the gold standard for the diagnosis of cervical lesions, histology specimens are more accurate than cervical smears for assessing cervical lesion status. Some studies assessed the methylation status of histological samples [2]. The conclusions had some differences among various studies. This is mainly related to the study of different target genes, detection methods and populations. These studies suggested that DNA methylation markers were associated with the severity of the lesion. Therefore, it is of clinical significance to explore the correlation between DNA methylation analysis and the degree of cervical lesions.

Various degrees of cervical epithelial lesions can be seen in the cervix of CIN patients. The DNA methylation marker panel of multiple tumor suppressor genes may improve the diagnostic accuracy of CIN and cancer. A methylation signature comprising the 5' regions of the genes *DLX1, ITGA4, RXFP3, SOX17* and *ZNF671* was specific for CIN3 + , with a specificity of 76.6% in women over 30 [16]. A further study found a DNA methylation panel composed of the above five genes and *ASTN1* showed higher specificity for CIN3 + than hrHPV-based screening [17]. An epidemiological study on the DNA hypermethylation marker panel consisting of the six marker regions demonstrated it can triage the hrHPV-positive population and reduce the referral rate of colposcopy [18]. The diagnostic test of DNA methylation comprising these six markers *(ASTN1, DLX1, ITGA4, RXFP3, SOX17,* and *ZNF671*) named GynTect® had been developed in the above studies. The results suggested that the GynTect® methylation assay may be of great value in identifying cervical precursor lesions, and providing immediate risk judgment for the progression of cervical lesions.

In this study, we observed the methylation levels of the marker regions *ASTN1*, *DLX1*, *ITGA4*, *RXFP3*, *SOX17* and *ZNF671* (GynTect® assay) in cervical precancerous and cancerous tissue. To more accurately judge the effect of methylation on cervical lesions, we compared the methylation levels of patients from two aspects. One was the methylation levels among cervical lesion patients with different degrees of lesions. The second was the methylation level between different lesion degrees in a cervical lesion patient.

## Methods

### Ethical approval

The study was approved by the Ethics Committee of the Second Xiangya Hospital of Central South University (ethics number LYF2021077). Informed consent forms were signed by all participants.

### Study design

One thousand three hundred sixty-two women undergoing colposcopy were invited to participate in the study at gynecological clinic of the Second Xiangya Hospital from July 2021 to March 2022. At the same time, we also collected cervical cancer patients in the inpatient department. The inclusion criteria were as follows: sexual life history; cervical cytology and hrHPV screening results; cervical lesions confirmed by histological results of colposcopy biopsy; no history of cervical surgery. The exclusion criteria were as follows: inconformity to the inclusion criteria (*n* = 168); unwillingness to participate in the study or inability to sign consents (*n* = 60); history of other malignancies (*n* = 45) and pregnancy (*n* = 22).

**Fig. 1:**
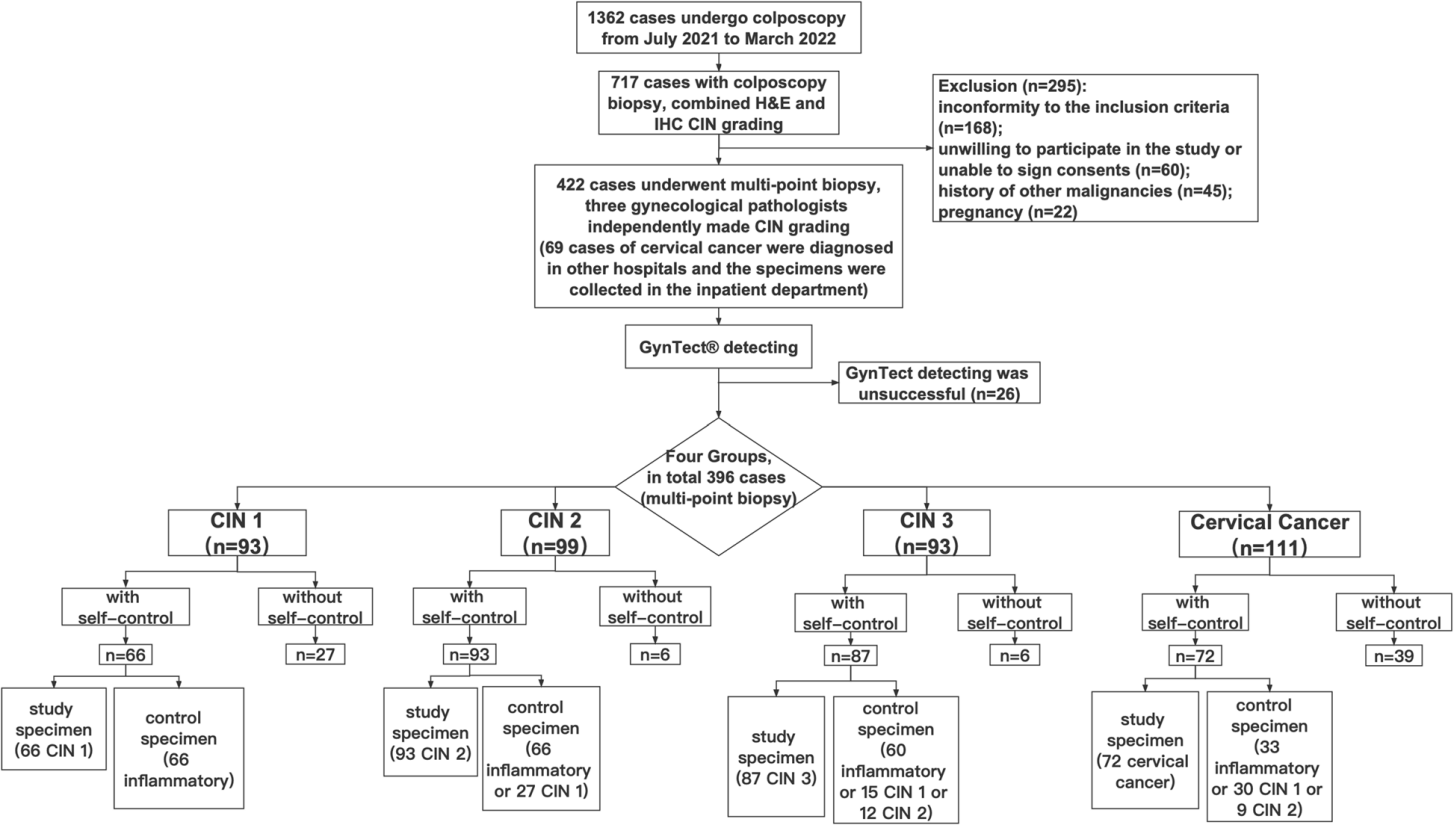
Flow chart of the study. CIN, cervical intraepithelial neoplasia; GynTect®, a diagnostic test of DNA methylation analysis of a methylation marker panel, the panel comprising six markers (*ASTN1, DLX1, ITGA4, RXFP3, SOX17*, and *ZNF671*)

The participants underwent multi-point biopsy and 422 cases met the research requirements. The methylation level of the DNA marker panel *(ASTN1*, *DLX1*, *ITGA4*, *RXFP3*, *SOX17* and *ZNF671)* were detected in cervical tissues of 422 cases. 26 cases were removed for unsuccessful methylation detection. Finally, 396 cases were available for analysis (Fig. 1). The included cases were 93 CIN1, 99 CIN2, 93 CIN3 and 111 cervical cancers.

We compared the methylation levels in patients with different cervical lesions and patients with the same cervical lesion. Patients with the same cervical lesions need paired pathological tissues for comparison. For example, the cervical biopsy samples of CIN1 patients for paired analysis included the study specimens (CIN1) and the control specimens (inflammatory or normal cervical tissue). By analogy, the control specimens of CIN2 or CIN3 samples for paired analysis was normal or inflammatory or CIN1 cervical tissue.

### Histopathology

The pathological diagnosis had been completed in the pathology department of our hospital. The diagnostic reference standard is the WHO classification of tumors of female reproductive organs and the LAST project in 2012 proposed using the immunohistochemical staining of p16 (p16 IHC) [19, 20]. The CIN diagnosis is based on H&E morphologic interpretation [21, 22]. According to a two-tier system, CIN1 is a low-grade squamous intraepithelial lesion (LSIL), and CIN2 or 3 are high-grade squamous intraepithelial lesions (HSIL). Three gynecological pathologists independently made CIN grades. Only 1% of patients still had inconsistent diagnoses for CIN grades after three gynecological pathologists commented together, and were excluded from the study.

P16INK4a immunohistochemistry (p16 IHC) has emerged as an adjunctive biomarker to aid in the evaluation of HSIL (CIN2) and in the distinction between HSIL and its mimics [23, 24]. Positive expression of P16 (MAB-0673) is defined as strong and continuous block-type staining of the nucleus and cytoplasm. Based on H&E morphological assessment, adding positive P16 results can provide objective evidence for HSIL.

### Thin-prep cytology test (TCT) and hrHPV detection

A liquid-based cervical cytology smear was taken with a cervical brush and immediately transferred to 20 mL of Thin-Prep PreservCyt Solution (Hologic, Beijing). The ThinPrep-2000 automatic cell detector was used for programmed processing. Cervical cytology diagnosis is classified into five levels according to the 2014 Bethesda System (TBS) criteria [25]. Abnormal TCT results are as follows: atypical squamous cells of undetermined significance (ASC-US), low-grade squamous intraepithelial lesion (LSIL), atypical squamous cells cannot exclude high-grade squamous intraepithelial lesion (ASC-H), high-grade squamous intraepithelial lesion (HSIL) and atypical glandular epithelial cells (AGC) within 3 months.

The Cervista HPV HR test (Hologic, Beijing) was an in vitro diagnostic test for the qualitative detection of 14 hrHPV DNA types (16, 18, 31, 33, 35, 39, 45, 51, 52, 56, 58, 59, 66, and 68). When hrHPV detection was positive, we used the Cervista HPV 16/18 test (Hologic, Beijing) to assess the presence or absence of hrHPV16 and 18.

### DNA extraction, bisulfite treatment and methylation-specific PCR (MSP) analysis

For performing the GynTect® assay (Oncgnostics GmbH, Jena, Germany), comprising six markers *ASTN1, DLX1, ITGA4, RXFP3, SOX17*, and *ZNF671*. Samples were processed as described in the instructions. The GynTect® assay was CE-certified for the use of STM™ (QIAGEN, Hilden, Germany) in 10/2015 [18]. DNA was extracted from frozen fresh cervical specimens by using the GynTect® Tissue Lysis Buffer Kit (Oncgnostics, Germany) and following the instructions of the manufacturer. After the cervical tissues were lysed, the EpiTect® Fast Bisulfite Kit (Qiagen, Germany) was used for purifying genomic DNA.

After elution of the bisulfite-converted DNA, a 10 μl sample was applied to each reaction system in the GynTect® real-time methylation-specific PCR (qMSP) assay with ABI7500 Real-Time PCR system (Life Technologies; Thermo Scientific, USA).

MSP program: 1 min period at 94℃; 42 cycles at 94℃ for 15 s (secs), 67℃ for 30 secs; followed by a melting curve analysis (95℃ for 15 secs, 60℃ for 20 secs, 95℃ for 30 secs, 30℃ for 60 secs). In each PCR reaction, 5 nanograms of genomic, bisulfite-treated DNA from tissue sections were used with 2.5 pmol of each methylation-specific primer in a total volume of 20 μl. Each marker produced a corresponding melting curve after 42 cycles, and the sample was considered qualified.

Acetylcholinesterase (*ACHE*) was used as the internal reference for DNA quality control, and the Ct value needs to be ≤ 36. Iduronate 2-sulfatase (*IDS*) was used as a quality control for methylation. The methylation-specific primers for *ACHE* and *ASTN1*, *DLX1*, *ITGA4*, *RXFP3*, *SOX17* and *ZNF671* were designed as previously described [18]. In addition, a positive control for determining the PCR quality was included in each PCR run.

Each marker had a Ct-value after the qMSP assay. The delta Ct (ΔCt) was calculated between the Ct-values of each marker and the quality control marker ACHE (ΔCt = Ct_(Marker)_-Ct_(ACHE)_). To be scored positive results, the ΔCt was defined as follows: ≤ 9 for 5 markers (*ASTN1, DLX1, ITGA4, RXFP3, SOX17)* and ≤ 10 for *ZNF671*. Each marker had a score after its ΔCt met the above positive criteria. The scores were as follows: *DLX1* 0.1; *ASTN1*, *ITGA4*, *RXFP3, SOX17* each 0.2; *ZNF671* 0.5. The positive GynTect® assay was the total score of six markers was ≥ 0.5.

### Statistical analysis

Statistical Package for Social Sciences (SPSS) version 23.0 was used for data analysis. All analyses were performed with a two-sided significance level of 0.05. The clinical characteristics of patients with different degrees of cervical lesions in four groups were compared. These clinical factors included the patient’s age, profession, household register, education level, marital status, fertility times, menopausal state, cytological abnormalities, hrHPV infection especially HPV16/18 infection and GynTect® positive rate. Logistic regression analysis was used to determine which factors were related to the severity of cervical lesions.

The Chi-square test or Fisher’s exact test (when at least one frequency < 5) was used to analyze the difference in the positive rate of methylation level of each host gene in cervical precancerous and cancer tissues. Chi-square segmentation was used to compare the differences between two groups. The methylation level differences of paired data including methylation score (paired t-test) and methylation positive rate (paired chi-square test) were further analyzed. The diagnostic value of the GynTect® assay for CIN2 or worse (CIN2 +) and CIN3 or worse (CIN3 +) was evaluated. The specificity, sensitivity, odds ratio (OR) and 95% confidence interval (CI) of different cervical cancer screening methods for CIN2 + and CIN3 + were calculated and analyzed.

## Results

### Clinical characteristics

Cervical tissue specimens from 396 women (median age 47.3 years) were available for analysis. The study population included 93 CIN1 (23.5%), 99 CIN2 (25.0%), 93 CIN3 (23.5%) and 111 cervical cancer (28.0%). Among them, 66 CIN1, 93 CIN2, 87 CIN3 and 72 cervical cancers were further used for paired analysis (Fig. 1).

The clinical characteristics of patients were grouped into four degrees of cervical lesions (CIN 1, CIN2, CIN3 and cervical cancer) for comparative analysis (Table 1 and Table S1). There were significant differences in demographic characteristics, such as age, profession, household register, education level, marital status, pregnancy times, fertility times and menopausal state among the four groups (all *P* < 0.05, Table S1). The results of cervical screening and the GynTect® assay had differences among cervical precancerous lesions and cervical cancer (all *P* < 0.05, Table 1). HPV16/18 infection and GynTect® positive results were more common in CIN2, CIN3 and cervical cancer groups.

**Table 1:** The results of cervical screening and GynTect® assay in cervical precancerous lesions and cervical cancer

	**Histological groups**				**Total**	***P*-value**
	**CIN 1**	**CIN 2**	**CIN 3**	**Cervical Cancer**		
**Cytology results(n)**						0.000*
Normal	42	40	17	17	116	
≥ ASC-US	51	59	74	81	265	
Unknown	0	0	2	13	15	
**hrHPV results(n)**						0.000*
16 and/or 18	18	41	44	76	179	
Other 12 hrHPV	49	42	3	16	152	
Unknown	6	9	92	10	28	
All 14 type	73	92		102	359	
**GynTect**® **assay(n)**						0.000*
Positive	45	66	75	111	297	
Negative	48	33	18	0	99	
Score (Mean ± SD)	0.72 ± 0.28	0.87 ± 0.36	1.13 ± 0.32	1.30 ± 0.20	1.11 ± 0.35	0.000*
**Score(n)**						0.000*
≥ 1.1	3	15	42	87	147	
< 1.1	90	84	51	24	249	

*CIN* Cervical intraepithelial neoplasia, *hrHPV* High-risk human papillomavirus, *hrHPV* Positive refers to the presence of one or more of the following HPV types HPV16, 18, 31, 33, 35, 39, 45, 51, 52, 56, 58, 59, 66, and 68, *SD* Standard deviation, *ASC-US* Atypical squamous cells of undetermined significance, *LSIL* Low-grade squamous intraepithelial lesion, *HSIL* High-grade squamous intraepithelial lesion, *GynTect®* A diagnostic test of DNA methylation analysis of a methylation marker panel, the panel comprising six markers (*ASTN1, DLX1, ITGA4, RXFP3, SOX17*, and *ZNF671*)

^*^, *P* < 0.05

### DNA marker methylation levels in cervical precancerous and cancerous lesions

We used GynTect® assay to detect the methylation levels of host genes in cervical precancerous lesions and cervical cancer. The positive rates of the GynTect® assay were 48.4% (45/93), 66.7% (66/99), 80.6% (75/93) and 100% (111/111) in CIN1, CIN2, CIN3 and cervical cancer, respectively (Table 1). There were significant differences among the four groups (*P* = 0.000). The scores of the GynTect® assay increased with the severity of cervical lesions, and significant differences were observed among four groups (*P* = 0.000). In particular, methylation levels with scores above the mean value (1.1) were more common in CIN2 + than in CIN1.

### Paired tests for DNA marker methylation

**Fig. 2:**
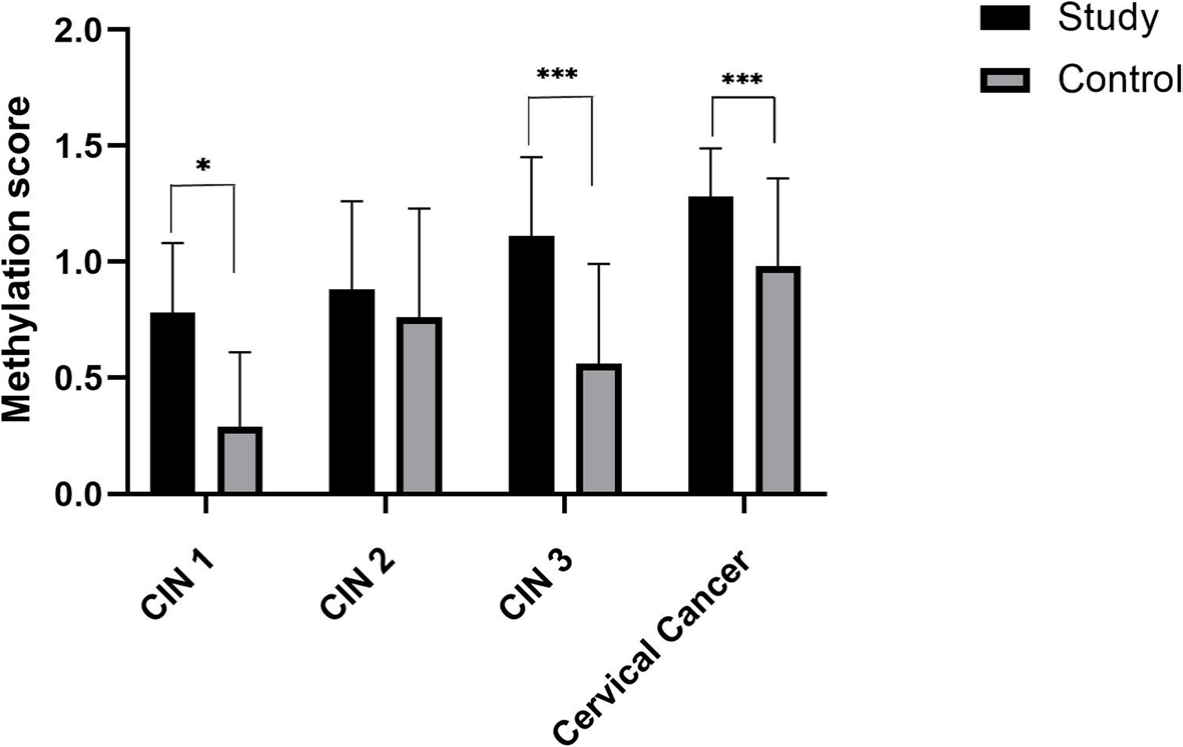
GynTect® assay results in paired-groups of cervical precancerous and cancerous lesions. CIN, cervical intraepithelial neoplasia; The mean scores of GynTect® in paired-groups (study specimens vs control specimens) were (CIN1, 0.78 ± 0.30 vs 0.30 ± 0.32), (CIN2, 0.88 ± 0.38 vs 0.76 ± 0.47), (CIN 3, 1.11 ± 0.34 vs 0.56 ± 0.43), and (cervical cancer, 1.28 ± 0.21 vs 0.98 ± 0.38), respectively. *P = 0.033, ***P = 0.000

We further analyzed the GynTect® results in paired-groups (Fig. 2). In each group, each pair of specimens included a study specimen and a control specimen. The severity of cervical lesions in the control specimens was lower than that in the study specimens. The mean scores of the GynTect® assay in paired-groups (study specimens vs control specimens) were (0.78 ± 0.30 vs 0.30 ± 0.32), (0.88 ± 0.38 vs 0.76 ± 0.47), (1.11 ± 0.34 vs 0.56 ± 0.43), and (1.28 ± 0.21 vs 0.98 ± 0.38) in CIN1, CIN2, CIN 3 and cervical cancer, respectively. The results were showed significant differences of DNA methylation levels in the paired groups of CIN1, CIN3 and cervical cancer (*P* = 0.033, 0.000 and 0.000, respectively). However, there was no difference between the study specimens and the control specimens in CIN2 group (*P* = 0.171).

According to the positive criteria of GynTect® (the sum scores of six host genes ≥ 0.5), the positive rate of GynTect® in each group had no difference between the study specimens and the control specimens (all *P* > 0.05, Table S2). This indicated that the methylation status of each point in the cervix can represent the cervix.

### DNA methylation levels of a single marker in the GynTect® assay

The GynTect® methylation assay contains six DNA methylation markers. We investigated the GynTect® positive rate of every marker in cervical precancerous and cancerous lesions (Table 2). The positive rate of every methylation marker by the GynTect® assay showed differences in four cervical lesion groups (all *P* < 0.05).

**Table 2:** The positive rate of every methylation marker in cervical precancerous and cancerous lesions

	**The positive rate (n)**				***P*-value**
Methylation marker	**CIN 1**	**CIN 2**	**CIN 3**	**Cervical Cancer**	
*ASTN1*	51.6%(48)	72.7%(72)	80.6%(75)	89.2%(99)	0.005*
*DLX1*	58.1%(54)	78.8%(78)	96.8%(90)	94.6%(105)	0.000*
*ITGA4*	6.5%(6)	12.1%(12)	32.3%(30)	75.7%(84)	0.000*
*RXFP3*	29.0%(27)	30.3%(30)	54.8%(51)	83.8% (93)	0.000*
*SOX17*	38.7%(36)	60.6%(60)	54.8%(51)	86.5%(96)	0.001*
*ZNF671*	12.9%(12)	30.3%(30)	71.0%(66)	97.3%(108)	0.000*
GynTect®	48.4%(45)	66.7%(66)	80.6%(75)	100.0%(111)	0.000*

*CIN* Cervical intraepithelial neoplasia, ASTN1 Astrotactin 1, *DLX1* Distal-less homeobox 1, *ITGA4* Integrin subunit alpha 4, *RXFP3* Relaxin family peptide receptor 3, *SOX17* SRY-box transcription factor 17, *ZNF671* Zinc finger protein 671, GynTect®, a diagnostic test of DNA methylation analysis of a methylation marker panel, the panel comprising six markers (*ASTN1, DLX1, ITGA4, RXFP3, SOX17*, and *ZNF671*)

^*^, *P* < 0.05

The three markers with the lowest positive rate of DNA methylation in CIN1 patients were *ITGA4* (6.5%)*, ZNF671* (12.9%) and *RXFP3* (29.0%). As the degree of cervical lesions rises, the positive methylation rate of *ZNF671* reached 30.3% in CIN2, 71.0% in CIN3 and 97.3% in cervical cancer (Table 2). The positive expression rates of *ZNF671* in cervical cancer and CIN3 were significantly higher than those in CIN1 (Table S3, *P* = 0.000, 0.000). The methylation positive rates of *RXFP3* and *ITGA4* in cervical cancer were significantly higher than those in CIN1.

The positive rate of GynTect® in cervical cancer and CIN3 showed similar changes in the comparison of the two groups (*P* = 0.016, CIN3 vs CIN1; *P* = 0.000, cervical cancer vs CIN1). The positive rate of *ZNF671* in CIN2 was significantly lower than that in CIN3 (*P* = 0.002). However, the positive rate of GynTect® between CIN2 and CIN3 was no different (*P* = 0.263).

### The diagnostic value of GynTect® in cervical lesions

For the diagnosis of CIN2 + /CIN3 + , the sensitivity and specificity of GynTect® assay were 83.2%/91.2% and 51.6%/42.2% respectively (Table 3). The sensitivity and specificity of hrHPV detecting for CIN2 + /CIN3 + were 95.0%/95.6% and 21.5%/14.1% respectively. The sensitivity and specificity of cytology for CIN2 + /CIN3 + were 74.3%/82.0% and 45.2%/42.7% respectively. The sensitivity and specificity of hrHPV and GynTect® for CIN2 + /CIN3 + were 78.1%/87.6%, 62.4%/51.6%. The specificity of GynTect® assay or hrHPV combined with GynTect® for CIN2 + /CIN3 + were higher than those of hrHPV test.

**Table 3:** Diagnosis performance of different test systems for detection of CIN2 + and CIN3 +

**Diagnosis performance**	**GynTect**	**hrHPV** **(all 14 types)**	**hrHPV** **(HPV 16/18)**	**Cytology**	**hrHPV and GynTect**
**Sensitivity % (95%CI)**					
CIN2 +	83.2 (74.1–89.6)	95.0 (91.7–97.1)	59.4 (52.3–66.2)	74.3 (68.8–79.2)	78.1 (72.9–82.5)
CIN3 +	91.2 (81.1–96.4)	95.6 (91.2–97.9)	53.5 (47.7–59.2)	82.0 (75.6–87.1)	87.6 (82.1–91.7)
**Specificity % (95%CI)**					
CIN2 +	51.6 (33.4–69.4)	21.5 (13.9–31.5)	69.3 (62.2–75.6)	45.2 (34.9–55.8)	62.4 (51.7–72.0)
CIN3 +	42.2 (30.2–55.2)	14.1 (9.6–20.0)	80.6 (70.9–87.8)	42.7 (35.7–50.0)	51.6 (44.3–58.8)

*CIN2 +*  Cervical intraepithelial neoplasia grade 2 and worse, *CIN3 +*  Cervical intraepithelial neoplasia grade 3 and worse, *CI* Confidence interval, *GynTect®* A diagnostic test of DNA methylation analysis of a methylation marker panel, the panel comprising six markers (*ASTN1, DLX1, ITGA4, RXFP3, SOX17*, and *ZNF671*), *hrHPV* High-risk human papillomavirus

The results showed that, among the six methylation markers, the marker with the highest sensitivity for detection of CIN2 + /CIN3 + was *DLX1*, followed by *ASTN1*, and *ITGA4* was the lowest sensitivity marker (Table S4). The specificity of *ITGA4* for diagnostic CIN2 + /CIN3 + was the highest, followed by *ZNF671* and *SOX17*.

The OR and 95% CI of positive ratios for detection of CIN2 + and CIN3 + by different test systems were listed in Table 4. With CIN1 as the reference, significantly higher ratios for the positive status of GynTect® were found in CIN2 + (OR 5.271, 95% CI 3.178–8.741, *P* = 0.000) and in CIN3 + (OR 11.022, 95% CI 5.858–20.738, *P* = 0.000), respectively. HrHPV test had significantly higher ratios in CIN2 + (OR 4.146, 95% CI 1.762–9.755, *P* = 0.001) and in CIN3 + (OR 10.462, 95% CI 4.356–25.125, *P* = 0.000), respectively. Cytology had significantly higher ratios in CIN2 + (OR 3.096, 95% CI 1.781–5.381, *P* = 0.000) and in CIN3 + (OR 5.438, 95% CI 3.101–9.536, *P* = 0.000), respectively. *ZNF671* methylation status had significantly higher ratios in CIN2 + (OR 13.909, 95% CI 7.246–26.698, *P* = 0.000) and in CIN3 + (OR 39.150, 95% CI 19.066–80.390, *P* = 0.000). With CIN1 as the reference, the remaining five single markers also had significantly higher ratios of positive methylation status in CIN2 + and CIN3 + (ORs arrange 3.297 ~ 10.322, 4.083 ~ 18.367, all *P* values = 0.000, Table S5).

**Table 4:** Positive ratios of different test systems for detection of CIN2 + and CIN3 +

**Histological result**	**GynTect®** **OR** **(95% CI)**	***P*-value**	**hrHPV** **OR** **(95% CI)**	***P*-value**	**Cytology** **OR** **(95% CI)**	***P*-value**	***ZNF671* methylation OR** **(95% CI)**	***P*-value**
CIN 1 (*n* = 93)	Reference		Reference		Reference		Reference	
CIN2 + (*n* = 303)	5.271 (3.178–8.741)	0.000*	4.146 (1.762–9.755)	0.001*	3.096 (1.781–5.381)	0.000*	13.909 (7.246–26.698)	0.000*
CIN3 + (*n* = 204)	11.022 (5.858–20.738)	0.000*	10.462 (4.356–25.125)	0.000*	5.438 (3.101–9.536)	0.000*	39.150 (19.066–80.390)	0.000*

*CIN* Cervical intraepithelial neoplasia, *CIN2 + * Cervical intraepithelial neoplasia grade 2 and worse, *CIN3 + * Cervical intraepithelial neoplasia grade 3 and worse, *GynTect®* A diagnostic test of DNA methylation analysis of a methylation marker panel, the panel comprising six markers (*ASTN1, DLX1, ITGA4, RXFP3, SOX17*, and *ZNF671*), *OR* Odds ratio, *CI* Confidence interval, *hrHPV* High-risk human papillomavirus, *ZNF671* Zinc finger protein 671

^*^, *P* < 0.05

## Discussion

Primary hrHPV-based screening provides sufficient sensitivity to detect high grade CIN and cancer, but low specificity brings excessive clinical workload and colposcopy referrals [26, 27]. Our results showed the methylation rate and score of six genes *ASTN1*, *DLX1*, *ITGA4*, *RXFP3*, *SOX17* and *ZNF671* increased with the severity of the lesion. The methylation scores above the mean value were more common in CIN2 + than in CIN1. The specificity of the DNA methylation marker panel was higher than that of the positive hrHPV test (51.6% vs 21.5% for CIN2 +). Women with positive methylation rate were 5.271 times and 11.022 times more likely to be CIN2 + and CIN3 + than those with negative methylation rate. Our study provides a basis for DNA methylation marker panel combined with primary hrHPV screening to identify the immediate risk of advanced CIN and cancer.

The characteristics of our study were to directly observe the methylation expression of a six-marker panel in pathological tissue specimens. The specimens were obtained by colposcopy biopsy, and each specimen site had corresponding pathological results. Therefore, the results of methylation conditions can well reflect the severity of cervical lesions. This would help us find the connection between DNA methylation of the six-marker panel and cervical precursor lesions. The same methylation panel in other four studies focused on triage hrHPV-positive women [16–18, 28]. Three studies collected cervical cell scrapes and one study used dry self-collected cervico-vaginal samples.

The first study in 2014 confirmed that the specificity of a methylation marker panel (*DLX1, ITGA4, RXFP3, SOX17* and *ZNF671*) for CIN3 + detection was 76.6% (95% CI: 65.6–85.5), and the usefulness of the five markers for triage after primary hrHPV testing [16]. The second study in 2017 evaluated the performance of GynTect® testing comprising six methylation markers as a triage test. The sensitivity of GynTect® was 67.7% (95% CI: 57.3–77.1) for CIN3 + detection, and the specificity of HPV-positive women in addition GynTect® testing reached the maximum value of 88.7% (95% CI: 83.7–92.6) [18]. The third study demonstrated the specificity of GynTect® for CIN3 + was 94.6% by using liquid-based cervical scrapes, compared to 69.9% for CINtec Plus and 82.6% for HPV and 90.6% for HPV 16/18 [17]. The fourth study confirmed the six-methylation-marker assay had a satisfactory amount of valid results on self-collected cervico-vaginal samples [28].

The methylation marker panel was used in cervical screening in the above four studies. Primary hrHPV screening had high sensitivity but with low specificity for cervical cancer screening. To prevent unnecessary referral, hrHPV-positive women required novel triage tests to improve specificity. Our study showed the specificity of hrHPV testing for CIN2 + and CIN3 + was lower than that of GynTect® assay, only 21.5% (95% CI: 13.9–31.5) and 14.1% (95% CI: 9.6–20.0). The specificity of the GynTect® assay on histological cervical specimens for CIN2 + and CIN3 + were 51.6% (95% CI: 33.4–69.4) and 42.2% (95% CI: 30.2–55.2). And the specificity of hrHPV testing and GynTect® assay reached 62.4% (95% CI: 51.7–72.0) and 51.6% (95% CI: 44.3–58.8). Therefore, the combination of hrHPV test and GynTect® assay improved the specificity of CIN diagnosis.

Our study found an interesting phenomenon. We found the positive rate of GynTect® in each paired group had no difference between the study specimens and the control specimens (such as CIN1, CIN2, CIN3 and cervical cancer, all *P* > 0.05, Table S2). This indicated that the methylation level of each point in the cervix can represent the cervix. The previous four studies of the GynTect® assay were all about cervical cancer screening. The specimen was obtained by cervical scraping in the four studies. Although the specimens were not obtained by point to point biopsy under colposcopy, as shown in our study, we speculated the GynTect® assay of cervical scrapes, just like the GynTect® assay of cervical tissue, can reflect the methylation condition of the cervix.

The study confirmed that the positive rate of the GynTect® assay was correlated with the severity of cervical lesions. Especially the methylation of *ZNF671* in CIN3 was significantly higher than that in CIN2 (71.0% vs 30.3%). And 97.3% of cervical cancer patients showed *ZNF671* DNA methylation. This rate was the highest among the six markers of cervical cancer. Other real-time methylation-specific PCR analyses for the *ZNF671* marker showed the highest detection rates for cervical scrapes with underlying CIN3 (67%) and cancer (90%) [16].

HSIL (CIN2 and CIN3) and cervical cancer are highly correlated with persistent hrHPV infection [29, 30]. HSIL is a heterogeneous disease and part of HSIL belongs to productive CIN. However, productive CIN is morphologically indistinguishable from transforming CIN [2]. The results of our study provide immediate risk judgment for the progression of cervical precursor lesions and cancer. The specificity of *ITGA4* and *ZNF671* methylation for CIN2 + reached 93.5% and 87.1% respectively. Patients with positive methylation rates of *ITGA4* and *ZNF671* genes were 10.322 times and 13.909 times more likely to have CIN2 + than those with negative methylation rates. Survival analysis established that the downregulation of *ZNF671* predicted poor prognosis in cervical squamous cell carcinoma [31]. The methylation score of *ZNF671* genes played an important proportion in the positive judgment of the GynTect® assay. Therefore, *ZNF671* was particularly useful in detecting CIN by the GynTect® assay. Another study confirmed that *ZNF671* and *DLX1* were the most predictive markers for the detection of CIN2 + in self-collected cervical samples [28].

DNA methylation of host genes and HPV-related genes in cervical epithelium played an important role in the diagnosis and treatment of cervical lesions [8, 11, 32–34]. In this study, we determined the value of DNA methylation marker panel (*ASTN1, DLX1, ITGA4, RXFP3, SOX17* and *ZNF671*) in detection of CIN lesions on cervical biopsy specimens. Our results demonstrate that the GynTect® has good diagnostic performance for detecting CIN2 + / CIN3 + when using alone or in combination with hrHPV.

The study had several shortcomings. The first was that all patients were from a single center and some selection bias may have occurred. The second was that the function of methylated genes had not been studied. We only detected the methylation status of six genes. The protein expressions of genes in tissues and the hypermethylation mechanism were unclear. At last, we had no follow-up data for patients. The main reason was most of CIN2 + patients in the study received surgical treatment, and we did not conduct individualized treatment according to the risks provided by methylation status. These limitations may be resolved in future trials.

## Conclusions

In conclusion, this study found that the promoter methylation of six tumor suppressor genes is related to the severity of cervical lesions. GynTect® test (comprising these six markers: *ASTN1, DLX1, ITGA4, RXFP3, SOX17* and *ZNF671*) has good diagnostic values for detecting CIN2 + and CIN3 + . The GynTect® assay based on cervical specimens can be used to identify advanced CIN and cervical cancer.

## Supplementary Information

**Additional file 1:** **Supplementary Figure 1.** The detailed comparisons of study design. CIN, cervical intraepithelial neoplasia; GynTect®, a diagnostic test of DNA methylation analysis of a methylation marker panel, the panel comprising six markers (*ASTN1, DLX1, ITGA4, RXFP3, SOX17*, and* ZNF671*). CIN2+, cervical intraepithelial neoplasia grade 2 and worse; CIN3+, cervical intraepithelial neoplasia grade 3 and worse.

**Additional file 2:** **Table S1**. The demographic characteristics of patients in cervical precancerous lesions and cervical cancer.

**Additional file 3:** **Table S2.** The results of GynTect® methylation assay in paired cervical precancerous and cervical cancer.

**Additional file 4:** **Table S3.** Intra-group comparison of GynTect® and* ZNF671 *methylation positive rate in cervical precancerous and cancerous lesions.

**Additional file 5:** **Table S4.** Diagnosis performance of single methylation marker in GynTect® assay for detection of CIN2+ and CIN3+

**Additional file 6:** **Table S5.** Positive ratios of a single marker for detection of CIN2+ and CIN3+ in GynTect® assay.

## Data Availability

All data for this study are contained in the supplement file.

## Abbreviations

ASC-US: Atypical squamous cells of undetermined significance
*ASTN1*: Astrotactin 1
*CADM1*: Cell adhesion molecule 1
*CDH1*: Cadherin 1
CIN: Cervical intraepithelial neoplasia
CIN2 +: Cervical intraepithelial neoplasia grade 2 and worse
CIN3 +: Cervical intraepithelial neoplasia grade 3 and worse
CI: Confidence interval
Ct value: Cycle threshold value
*DAPK1*: Death associated protein kinase 1
*DLX1*: Distal-less homeobox 1
DNA: Deoxyribonucleic acid
*EPB41L3*: Erythrocyte membrane protein band 4.1 like 3
*FAM19A4*: Family with sequence similarity 19 member A4
GynTect®: A diagnostic test of DNA methylation analysis of a methylation marker panel, the panel comprising six markers (*ASTN1*, *DLX1*, *ITGA4*, *RXFP3*, *SOX17* and *ZNF671*)
hrHPV: High-risk human papillomavirus
hrHPV: Positive refers to the presence of one or more of the following HPV types HPV16, 18, 31, 33, 35, 39, 45, 51, 52, 56, 58, 59, 66 and 68
HSIL: High-grade squamous intraepithelial lesion
*ITGA4*: Integrin subunit alpha 4
LSIL: Low-grade squamous intraepithelial lesion
*MAL*: Mal, T cell differentiation protein
qMSP: Real-time methylation-specific PCR
OR: Odds ratio
*PAX1*: Paired box 1
PhD: Philosophic doctor
*PRDM14*: PR/SET domain 14; *RXFP3,* relaxin family peptide receptor 3
SD: Standard deviation
*SOX17*: SRY-box transcription factor 17
*TERT*: Telomerase reverse transcriptase and *ZNF671,* zinc finger protein 671

## References

[1] Bray F (2018). Global cancer statistics 2018: GLOBOCAN estimates of incidence and mortality worldwide for 36 cancers in 185 countries. CA Cancer J Clin.

[2] Del Pino M (2019). CADM1, MAL, and miR124 Promoter Methylation as Biomarkers of Transforming Cervical Intrapithelial Lesions. Int J Mol Sci.

[3] Steenbergen R (2014). Clinical implications of (epi)genetic changes in HPV-induced cervical precancerous lesions. Nat Rev Cancer.

[4] Vink F (2021). Classification of high-grade cervical intraepithelial neoplasia by p16, Ki-67, HPV E4 and FAM19A4/miR124-2 methylation status demonstrates considerable heterogeneity with potential consequences for management. Int J Cancer.

[5] Kremer W (2020). Characterization of cervical biopsies of women with HIV and HPV co-infection using p16, ki-67 and HPV E4 immunohistochemistry and DNA methylation. Mod Pathol.

[6] Fang J, Zhang H, Jin S (2014). Epigenetics and cervical cancer: from pathogenesis to therapy. Tumour Biol.

[7] Bowden S (2019). The use of human papillomavirus DNA methylation in cervical intraepithelial neoplasia: A systematic review and meta-analysis. EBioMedicine.

[8] Kremer W (2021). The use of host cell DNA methylation analysis in the detection and management of women with advanced cervical intraepithelial neoplasia: a review. BJOG.

[9] Verlaat W (2018). Host-cell DNA methylation patterns during high-risk HPV-induced carcinogenesis reveal a heterogeneous nature of cervical pre-cancer. Epigenetics.

[10] Bee K (2021). Genetic and Epigenetic Variations of HPV52 in Cervical Precancer. Int J Mol Sci.

[11] Huang S (2019). Association Study Between Methylation in the Promoter Regions of cGAS, MAVS, and TRAF3 Genes and the Risk of Cervical Precancerous Lesions and Cervical Cancer in a Southern Chinese Population. Front Genet.

[12] Liang H (2021). The application value of PAX1 and ZNF582 gene methylation in high grade intraepithelial lesion and cervical cancer. Clin Transl Oncol.

[13] Mersakova S (2016). DNA methylation and detection of cervical cancer and precancerous lesions using molecular methods. Tumour Biol.

[14] El Aliani A (2021). Association between Gene Promoter Methylation and Cervical Cancer Development: Global Distribution and A Meta-analysis. Cancer Epidemiol Biomarkers Prev.

[15] Kremer WW (2019). Role of FAM19A4/miR124-2 methylation analysis in predicting regression or non-regression of CIN2/3 lesions: a protocol of an observational longitudinal cohort study. BMJ Open.

[16] Hansel A (2014). A promising DNA methylation signature for the triage of high-risk human papillomavirus DNA-positive women. PLoS ONE.

[17] Schmitz M (2018). Performance of a DNA methylation marker panel using liquid-based cervical scrapes to detect cervical cancer and its precancerous stages. BMC Cancer.

[18] Schmitz M (2017). Performance of a methylation specific real-time PCR assay as a triage test for HPV-positive women. Clin Epigenetics.

[19] Darragh T (2013). The Lower Anogenital Squamous Terminology Standardization project for HPV-associated lesions: background and consensus recommendations from the College of American Pathologists and the American Society for Colposcopy and Cervical Pathology. Int J Gynecol Pathol.

[20] Kurman RJ, Carcangiu ML, Herrington CS, Young RH, editors. WHO Classification of tumours of female reproductive organs. 4th ed. Lyon: IARC Press; 2014.

[21] Lax S (2016). New features in the 2014 WHO classification of uterine neoplasms. Pathologe.

[22] Villegas-Hinojosa E (2020). Histopathological Diagnosis of Cervical Biopsies: Reduction of Sampling Errors with the Evaluation of a Third Histologic Level. Cancer Manag Res.

[23] Wu M (2019). The Diagnostic Utility of p16 Immunostaining in Differentiating Cancer and HSIL from LSIL and Benign in Cervical Cells. Cell Transplant.

[24] Arvizo C (2016). p16 Immunohistochemistry in Colposcope-Directed and Random Cervical Biopsies of CIN2 and CIN3. J Low Genit Tract Dis.

[25] Nayar R, Wilbur DC (2015). The Pap test and Bethesda 2014. Cancer Cytopathol.

[26] Liang LA (2021). Cervical Cancer Screening: Comparison of Conventional Pap Smear Test, Liquid-Based Cytology, and Human Papillomavirus Testing as Stand-alone or Cotesting Strategies. Cancer Epidemiol Biomarkers Prev.

[27] Kaljouw S (2021). Reducing unnecessary referrals for colposcopy in hrHPV-positive women within the Dutch cervical cancer screening programme: A modelling study. Gynecol Oncol.

[28] Klischke L (2021). Performance of a six-methylation-marker assay on self-collected cervical samples - A feasibility study. J Virol Methods.

[29] Porcari AM (2018). Molecular Signatures of High-Grade Cervical Lesions. Front Oncol.

[30] Bonde JH (2020). Clinical Utility of Human Papillomavirus Genotyping in Cervical Cancer Screening: A Systematic Review. J Low Genit Tract Dis.

[31] Zhang J (2019). ZNF671Epigenetic-Mediated Downregulation of Zinc Finger Protein 671 () Predicts Poor Prognosis in Multiple Solid Tumors. Front Oncol.

[32] Burley M, Roberts S, Parish J (2020). Epigenetic regulation of human papillomavirus transcription in the productive virus life cycle. Semi Immunopathol.

[33] Dick S (2020). Evaluation of six methylation markers derived from genome-wide screens for detection of cervical precancer and cancer. Epigenomics.

[34] Kelly H (2019). Performance of DNA methylation assays for detection of high-grade cervical intraepithelial neoplasia (CIN2+): a systematic review and meta-analysis. Br J Cancer.

