# Supplementary figures and images for: Promoter hypermethylation analysis of host genes in cervical intraepithelial neoplasia and cervical cancers on histological cervical specimens
Source: BMC Cancer. 2023 Feb 20;23:168. doi: 10.1186/s12885-023-10628-5 (PMC9940376; doi:10.1186/s12885-023-10628-5)

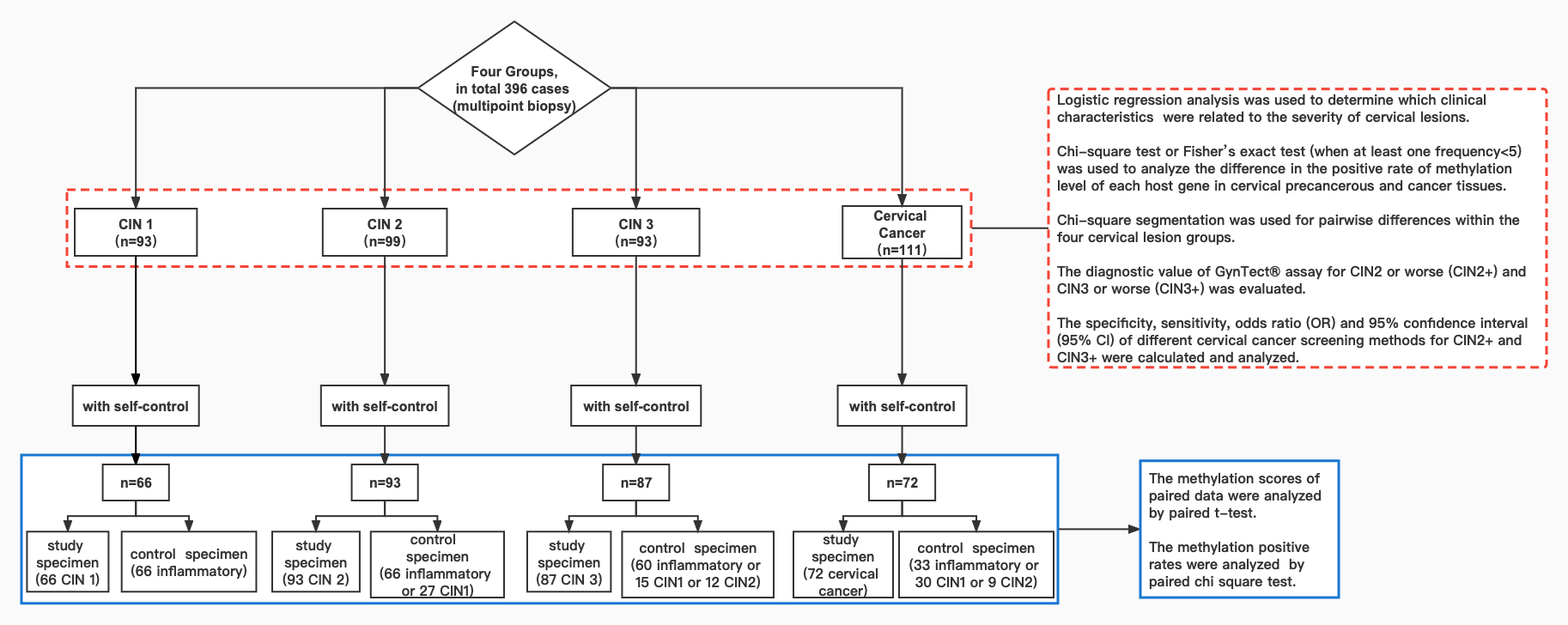

Supplement: Supplementary file 1 — Additional file 1: Supplementary Figure 1. The detailed comparisons of study design. CIN, cervical intraepithelial neoplasia; GynTect®, a diagnostic test of DNA methylation analysis of a methylation marker panel, the panel comprising six markers (ASTN1, DLX1, ITGA4, RXFP3, SOX17, and ZNF671). CIN2+, cervical intraepithelial neoplasia grade 2 and worse; CIN3+, cervical intraepithelial neoplasia grade 3 and worse. [file 12885_2023_10628_MOESM1_ESM.bmp]
