# Supplementary material for: Promoter hypermethylation analysis of host genes in cervical intraepithelial neoplasia and cervical cancers on histological cervical specimens
Source: BMC Cancer. 2023 Feb 20;23:168. doi: 10.1186/s12885-023-10628-5 (PMC9940376; doi:10.1186/s12885-023-10628-5)
Supplement: Supplementary file 2 — Additional file 2: Table S1. The demographic characteristics of patients in cervical precancerous lesions and cervical cancer. [file 12885_2023_10628_MOESM2_ESM.docx]

**Table S1** The demographic characteristics of patients in cervical precancerous lesions and cervical cancer

| **Parameters** | **Histological groups** | | | | **Total** | ***P*-value** |
| --- | --- | --- | --- | --- | --- | --- |
|  | **CIN 1** | **CIN 2** | **CIN 3** | **Cervical Cancer** |  |  |
| **Number (%)** | 93(23.5) | 99(25.0) | 93 (23.5) | 111(28.0) | 396(100.0) |  |
| **Age(Mean ± SD, years)** | 47.5±11.5 | 41.5±10.2 | 44.9±10.7 | 52.9±9.4 | 47.3±11.3 | 0.000* |
| **Profession**  Housewife  office worker  Unknown | 37  54  2 | 42  56 1 | 46  47  0 | 79  32  0 | 204  189  3 | 0.000* |
| **Household register**  Countryside  Town | 23  70 | 24  75 | 45  48 | 55  56 | 147  249 | 0.000* |
| **Education level**  Primary  Secondary  College  Master or PhD | 2  61  28  2 | 6  55  36 2 | 3  66  24  0 | 18  75  18  0 | 29  257  106  4 | 0.000* |
| **Marital status**  Unmarried  Married  Divorce | 8  85  0 | 13  86  0 | 3  86  4 | 0  107  4 | 24  364  8 | 0.000* |
| **Pregnancy times**  0  1-2  ＞2 | 5  26  62 | 12  35 52 | 4  33  56 | 0  33  78 | 21  127  248 | 0.002 |
| **Fertility times**  0  1-2  ＞2 | 9  72  12 | 14  72 13 | 8  72  13 | 2  80  29 | 33  296  67 | 0.005* |
| **Menopausal state**  Yes  No | 67  26 | 88  11 | 74  19 | 52  59 | 281  115 | 0.000* |

*: *P*＜0.05

CIN, cervical intraepithelial neoplasia; SD, standard deviation; PhD, philosophic doctor.
