# Supplementary material for: Promoter hypermethylation analysis of host genes in cervical intraepithelial neoplasia and cervical cancers on histological cervical specimens
Source: BMC Cancer. 2023 Feb 20;23:168. doi: 10.1186/s12885-023-10628-5 (PMC9940376; doi:10.1186/s12885-023-10628-5)
Supplement: Supplementary file 3 — Additional file 3: Table S2. The results of GynTect® methylation assay in paired cervical precancerous and cervical cancer. [file 12885_2023_10628_MOESM3_ESM.docx]

**Table S2** The results of GynTect® methylation assay in paired cervical precancerous and cervical cancer

| **Histological paired groups(n)** | **GynTect® assay(n)** | | ***P*-value** |
| --- | --- | --- | --- |
|  | **positive** | **negative** |  |
| **CIN 1(n=66)** |  |  | **0.353** |
| study specimens  (66 CIN 1) | 30 | 36 |  |
| control specimens  (66 inflammatory) | 21 | 45 |  |
| **CIN 2(n=93)** |  |  | **0.303** |
| study specimens  (93 CIN 2) | 60 | 33 |  |
| control specimens  (66 inflammatory or 27 CIN1) | 48 | 45 |  |
| **CIN 3(n=87)** |  |  | **0.162** |
| study specimens  (87 CIN 3) | 66 | 21 |  |
| control specimens  (60 inflammatory or 15 CIN1 or 12 CIN2) | 51 | 30 |  |
| **Cervical Cancer (n=72)** |  |  | **0.489** |
| study specimens  (72 cervical cancer) | 72 | 0 |  |
| control specimens  (33 inflammatory or 30 CIN1 or 9 CIN2) | 66 | 6 |  |

*: *P*＜0.05

CIN, cervical intraepithelial neoplasia; GynTect®, a diagnostic test of DNA methylation analysis of a methylation marker panel, the panel comprising six markers (*ASTN1, DLX1, ITGA4, RXFP3, SOX17*, and *ZNF671*).
