# Supplementary material for: Promoter hypermethylation analysis of host genes in cervical intraepithelial neoplasia and cervical cancers on histological cervical specimens
Source: BMC Cancer. 2023 Feb 20;23:168. doi: 10.1186/s12885-023-10628-5 (PMC9940376; doi:10.1186/s12885-023-10628-5)
Supplement: Supplementary file 4 — Additional file 4: Table S3. Intra-group comparison of GynTect® and ZNF671 methylation positive rate in cervical precancerous and cancerous lesions. [file 12885_2023_10628_MOESM4_ESM.docx]

**Table S3**  Intra-group comparison of GynTect® and *ZNF671* methylation positive rate in cervical precancerous and cancerous lesions

|  | **Positive rates (n/total)** | | ***P*-value** |
| --- | --- | --- | --- |
| **Histological groups** | **control group** | **study group** |  |
| **CIN 1 vs CIN 2**  GynTect®  *ZNF671* methylation | 48.3% (45/93)  12.9% (12/93) | 66.7% (66/99)  30.3% (30/99) | 0.205  0.132 |
| **CIN 1 vs CIN 3**  GynTect®  *ZNF671* methylation | 48.3% (45/93)  12.9% (12/93) | 80.6% (75/93)  71.0% (66/93) | 0.016*  0.000* |
| **CIN 1 vs cervical cancer**  GynTect®  *ZNF671* methylation | 48.3% (45/93)  12.9% (12/93) | 100.0% (111/111)  97.3% (108/111) | 0.000*  0.000* |
| **CIN 2 vs CIN 3**  GynTect®  *ZNF671* methylation | 66.7% (66/99)  30.3% (30/99) | 80.6% (75/93)  71.0% (66/93) | 0.263  0.002* |
| **CIN 2 vs cervical cancer**  GynTect®  *ZNF671* methylation | 66.7% (66/99)  30.3% (30/99) | 100.0% (111/111)  97.3% (108/111) | 0.000*  0.000* |
| **CIN 3 vs cervical cancer**  GynTect®  *ZNF671* methylation | 80.6% (75/93)  71.0% (66/93) | 100.0% (111/111)  97.3% (108/111) | 0.007*  0.004* |

*: *P*＜0.05

CIN, cervical intraepithelial neoplasia; *ZNF671*, zinc finger protein 671; GynTect®, a diagnostic test of DNA methylation analysis of a methylation marker panel, the panel comprising six markers (*ASTN1, DLX1, ITGA4, RXFP3, SOX17*, and *ZNF671*).
