# Supplementary material for: Promoter hypermethylation analysis of host genes in cervical intraepithelial neoplasia and cervical cancers on histological cervical specimens
Source: BMC Cancer. 2023 Feb 20;23:168. doi: 10.1186/s12885-023-10628-5 (PMC9940376; doi:10.1186/s12885-023-10628-5)
Supplement: Supplementary file 5 — Additional file 5: Table S4. Diagnosis performance of single methylation marker in GynTect® assay for detection of CIN2+ and CIN3+ [file 12885_2023_10628_MOESM5_ESM.docx]

**Table** **S4** Diagnosis performance of single methylation marker in GynTect® assay for detection of CIN2+ and CIN3+

| **Diagnosis performance** | ***ZNF671*** | ***SOX17*** | ***RXFP3*** | ***ITGA4*** | ***DLX1*** | ***ASTN1*** |
| --- | --- | --- | --- | --- | --- | --- |
| **Sensitivity %**  **(95%CI)** |  |  |  |  |  |  |
| CIN2+ | 67.3  (57.2-76.1) | 68.3  (58.2-77.0) | 57.4  (47.2-67.1) | 41.6  (32.0-51.8) | 90.1  (82.1-94.9) | 81.2  (71.9-88.0) |
| CIN3+ | 85.3  (74.2-92.3) | 72.1  (59.7-81.9) | 70.6  (58.1-80.7) | 55.9  (43.4-67.7) | 95.6  (86.8-98.9) | 85.3  (74.2-92.3) |
| **Specificity %**  **(95%CI)** |  |  |  |  |  |  |
| CIN2+ | 87.1  (69.2-95.8) | 61.3  (42.3-77.6) | 71.0  (51.8-85.1) | 93.5  (77.2-98.9) | 41.9  (25.1-60.7) | 48.4  (30.6-66.6) |
| CIN3+ | 78.1  (65.7-87.1) | 50.0  (37.3-38.8) | 70.3  (57.4-80.8) | 90.6  (80.1-96.1) | 31.3  (20.6-44.2) | 37.5  (26.0-50.5) |

GynTect®, a diagnostic test of DNA methylation analysis of a methylation marker panel, the panel comprising six markers (*ASTN1, DLX1, ITGA4, RXFP3, SOX17*, and *ZNF671*); CIN2+, cervical intraepithelial neoplasia grade 2 and worse; CIN3+, cervical intraepithelial neoplasia grade 3 and worse; CI: confidence interval; PPV, positive predictive value; NPV, negative predictive value; *ASTN1*, astrotactin 1; *DLX1*, distal-less homeobox 1; *ITGA4*, integrin subunit alpha 4; *RXFP3*, relaxin family peptide receptor 3; *SOX17*, SRY-box transcription factor 17; *ZNF671*, zinc finger protein 671.
