# Supplementary material for: Promoter hypermethylation analysis of host genes in cervical intraepithelial neoplasia and cervical cancers on histological cervical specimens
Source: BMC Cancer. 2023 Feb 20;23:168. doi: 10.1186/s12885-023-10628-5 (PMC9940376; doi:10.1186/s12885-023-10628-5)
Supplement: Supplementary file 6 — Additional file 6: Table S5. Positive ratios of a single marker for detection of CIN2+ and CIN3+ in GynTect® assay. [file 12885_2023_10628_MOESM6_ESM.docx]

**Table S5** Positive ratios of a single marker for detection of CIN2+ and CIN3+ in GynTect® assay

| **Histological result** | ***ASTN1***  **OR**  **(95% CI)** | ***P*-value** | ***DLX1***  **OR**  **(95% CI)** | ***P*-value** | ***ITGA4***  **OR**  **(95% CI)** | ***P*-value** | ***RXFP3***  **OR**  **(95% CI)** | ***P-*value** | ***SOX17***  **OR**  **(95% CI)** | ***P*-value** |
| --- | --- | --- | --- | --- | --- | --- | --- | --- | --- | --- |
| **CIN1**  **(n=93)** | Reference |  | Reference |  | Reference |  | Reference |  | Reference |  |
| **CIN2+**  **(n=303)** | 4.046  (2.458-6.660) | 0.000* | 5.747  (3.275-10.085) | 0.000* | 10.322  (4.376-24.350) | 0.000* | 3.297  (1.995-5.449) | 0.000* | 3.524  (2.173-5.716) | 0.000* |
| **CIN3+**  **(n=204)** | 5.438  (3.101-9.536) | 0.000* | 13.684  (6.224-30.085) | 0.000* | 18.367  (7.678-43.937) | 0.000* | 5.867  (3.420-10.063) | 0.000* | 4.083  (2.434-6.850) | 0.000* |

*: *P*＜0.05

CIN, cervical intraepithelial neoplasia; CIN2+, cervical intraepithelial neoplasia grade 2 and worse; CIN3+, cervical intraepithelial neoplasia grade 3 and worse; GynTect®, a diagnostic test of DNA methylation analysis of a methylation marker panel, the panel comprising six markers (*ASTN1, DLX1, ITGA4, RXFP3, SOX17*, and *ZNF671*); OR, odds ratio; CI, confidence interval; *ASTN1*, astrotactin 1; *DLX1*, distal-less homeobox 1; *ITGA4*, integrin subunit alpha 4; *RXFP3*, relaxin family peptide receptor 3; *SOX17*, SRY-box transcription factor 17; *ZNF671*, zinc finger protein 671.
